# Risk of uterine leiomyoma based on BET1L rs2280543 single nucleotide polymorphism and vegetarian diet

**DOI:** 10.1186/s12905-022-01721-1

**Published:** 2022-04-27

**Authors:** Shan Chih Lee, Ying-Hsiang Chou, Disline Manli Tantoh, Shu-Yi Hsu, Oswald Ndi Nfor, Yeu Sheng Tyan, Yung-Po Liaw

**Affiliations:** 1 School of Medical Imaging and Radiological Sciences, Chung Shan Medical University, Taichung City, 40201 Taiwan; 2 Department of Radiation Oncology, Chung Shan Medical University Hospital, Taichung, 40201 Taiwan; 3 Institute of Medicine, Chung Shan Medical University, Taichung City, 40201 Taiwan; 4 Department of Medical Imaging, Chung Shan Medical University Hospital, Taichung City, 40201 Taiwan; 5 Department of Public Health and Institute of Public Health, Chung Shan Medical University, No. 110, Sec. 1 Jianguo N. Rd, Taichung City, 40201 Taiwan; 6 School of Medicine, Chung Shan Medical University, Taichung City, 40201 Taiwan; 7 School of Medical Informatics, Chung Shan Medical University, Taichung City, 40201 Taiwan; 8 Medical Imaging and Big Data Center, Chung Shan Medical University Hospital, Taichung City, 40201 Taiwan

**Keywords:** Uterine fibroid, BET1L rs2280543, Genotype, Vegetarian diet, Epidemiology

## Abstract

**Background:**

Bet1 Golgi vesicular membrane trafficking protein-like (BET1L) rs2280543 single nucleotide polymorphism (SNP) and diet have been independently associated with uterine leiomyoma (UL). However, whether the SNP and diet could jointly influence the risk of UL is yet to be assessed. Therefore, we investigated the independent and interactive effects of vegetarian diet and BET1L rs2280543 on uterine fibroids in Taiwanese women.

**Methods:**

We linked participants’ electronic data in the Taiwan Biobank (TWB) database to their medical records in the National Health Insurance Research Database (NHIRD). The TWB had genotypic, lifestyle, and biochemical data between 2008 and 2015 and the NHIRD had data on disease diagnoses between 1998 and 2015. In this study, we included 1997 premenopausal women with complete data.

**Results:**

Compared  to participants with the BET1L rs2280543 CC genotype (wildtype), those with CT/CC genotype had an odds ratio (OR) of 0.69 and a 95% confidence interval (CI) of 0.51–0.93. Vegetarian diet and UL were not significantly associated: OR = 1.09 and 95% CI = 0.77–1.55. However, the test for interaction between rs2280543 and vegetarian diet was significant (*p* = 0.046). Compared to individuals with the CC genotype, the risk of UL was lower among vegetarians with the CT/TT genotype: OR (95% CI) = 0.15 (0.05–0.47).

**Conclusion:**

The BET1L rs2280543 CT/TT genotype was associated with a lower risk of UL especially among vegetarians.

**Supplementary Information:**

The online version contains supplementary material available at 10.1186/s12905-022-01721-1.

## Introduction

Uterine fibroids (UFs) or uterine leiomyomas (ULs) are benign tumors caused by excessive growth of the uterine smooth muscle and connective tissue [1]. They are associated with significant morbidity and poor quality of life [2]. Environmental and genetic factors contribute to the pathogenesis of UL [2–8]. Some of the non-genetic factors are diet, age, body mass index (BMI), smoking, and alcohol consumption [2, 4–6]. It has been suggested that dietary components that have estrogen-mimicking properties and regulate the metabolism of endogenous estrogen may contribute to the development of UL [9]. Vegetarian diet, which is low in fat but rich in fibers, antioxidants, vitamins, phytochemicals, and minerals can protect against UL [8, 10–14].

Genetic predisposition plays a significant role in the onset and course of UL [15]. However, most genetic determinants of UL are still being explored. Consequently, the epidemiology of the disease has not been fully established [3, 16–19]. In-depth knowledge of the genetics of UL could help in identifying susceptible individuals [7, 18, 20, 21]. Hence, elucidating the molecular mechanisms behind UL development is important.

Genetic susceptibility to UL has been identified through genome-wide association studies (GWAS) that have uncovered DNA variations associated with the condition [20–22]. Single nucleotide polymorphisms (SNPs) are the most common type of gene variants in humans [19]. GWAS have identified BET1L rs2280543 as one of the most significant candidate SNPs for UL [21, 23]. The association of BET1L rs2280543 polymorphism with UF was initially reported among Japanese women [21] and later replicated in Han Chinese [24], European American [18, 23], and Japanese women [25]. However, both variables did not appear to be significantly related among Saudi [26] and black American women [27].

Due to conflicting reports regarding UL predisposing factors [4, 27, 28], it is crucial to evaluate the etiology of the disease [2, 18]. Moreover, replication of identified UL-associated SNPs within different populations is needed to validate the findings from GWAS [29]. To our knowledge, the association between BET1L rs2280543 and UL has not been determined among Taiwanese women. Therefore, we conducted this study to investigate the relationship between BET1L rs2280543 and UL among Taiwanese premenopausal women. Because UL has a multifactorial etiology, we further investigated whether BET1L rs2280543 and vegetarian diet could interact and influence the risk of UL.

## Materials and methods

### Data source

Using encrypted personal identification numbers, we linked the TWB, a repository containing genotypic, lifestyle, and biochemical data collected between 2008 and 2015 to the NHIRD which had information on diseases diagnosed between 1998 and 2015. All analyses were done at the Health and Welfare Data Science Center (HWDC) [30].

### Variable definitions

In the NHIRD, we identified diseases based on either two outpatient visits or one-time admission using ICD-9 codes. The codes were 218.0, 218.1, 218.2, and 218.9 (for uterine fibroids), 401-405, A260, A269 (for hypertension), and 250, A181 (for diabetes mellitus). Vegetarians included those who maintained a vegetarian diet for at least six months prior to data collection. Participants who used western hormonal medications for more than half a year for gynecologic purposes like contraception, menopausal syndrome, and others were considered as hormone users. Herbal medicine users referred to those who used Chinese traditional medicine continuously for at least three months and were still using it during their recruitment into the Biobank project. Other variables such as physical activity, cigarette smoking, body mass index (BMI), alcohol, tea, and coffee consumption, have previously been described [31, 32]. We assessed and categorized fat intake based on twelve questions pertaining to the frequency of eating fat-containing foods over the previous month (Additional file 1: Table S1). The response scale ranged from 1 to 5 (i.e., 1 = never, 2 = seldom, 3 = sometimes, 4 = frequently, and 5 = always). We derived the fat intake scores (ranging from 12 to 60) for participants by calculating their responses to the 12 questions. The scores were grouped into quartiles: ≤ 35 (< Q1), 36–40 (Q1–Q2), 41–44 (Q2–Q3), and ≥ 45 (> Q3). The higher the score, the more likely the participants were inclined to low-fat foods.

BET1L rs2280543 was selected based on literature search and was genotyped using the custom Taiwan biobank chip (Axiom™ Genome-Wide Array Plate System (Affymetrix, Santa Clara, CA, USA). This SNP was included in the Taiwan Biobank because it met the quality control criteria: call rate > 95%, *p* value > 1.0 × 10^–3^ for the Hardy–Weinberg equilibrium test, and minor allele frequency > 0.05.

### Exclusion criteria

Initially, we included 2578 premenopausal women in the study. However, we excluded those with incomplete questionnaires (n = 578) and genotype (n = 3). Complete data were available for 341 cases of uterine fibroid (test group) and 1656 controls. Signed informed consents were obtained from all participants. We obtained ethical approval from the Institutional Review Board of Chung Shan Medical University Hospital (CS2-20006).

### Statistical analyses

All analyses were done using PLINK 1.09 and the statistical analysis system (SAS) software (version 9.4). We compared the differences between categorical and continuous variables using the Chi-square test and t-test, respectively. The test group comprised women with uterine fibroid, while the controls were those without uterine fibroid. Using logistic regression analysis, we estimated ORs for uterine fibroid with their 95% CI based on vegetarian status and rs2280543 genotypes. We also used logistic regression analysis to determine the interaction between vegetarian diet and BET1L rs2280543 on UF. Covariates included in the regression model were age, smoking, alcohol intake, exercise, hypertension, diabetes, hormone use, coffee and tea consumption, use of herbal medicine, family history of uterine fibroid, fat intake score, body mass index, parity, miscarriage, and or abortion.

## Results

Baseline characteristics of the 1997 participants stratified by vegetarian status are displayed in Table 1. There were 49 vegetarians and 292 non-vegetarians with uterine fibroids. BET1L rs2280543 genotypes, smoking, alcohol intake, exercise, hypertension, diabetes, hormone use, miscarriage/abortion, body mass index, age at menarche, parity, and family history of uterine fibroids did not differ between the two groups. However, age, coffee consumption, and fat intake scores between vegetarians and non-vegetarians differed significantly (*p* < 0.05). The rs2280543 CC genotype was the wildtype, C was the major allele, while T was the minor allele with a minor allele frequency (MAF) of 0.128935. Compared to participants with the BET1L rs2280543 CC genotype, those with the CT/TT genotype had a lower OR for UL (0.69; 95% CI 0.51–0.93), indicating a lower risk of getting the disease (Table 2). Vegetarians compared to non-vegetarians had an OR of 1.09 (95% CI 0.77–1.55). Compared to participants aged 30–39 years, those in the 40–49 and 50–59 age groups had ORs of 3.05 (95% CI 2.20–4.21) and 4.51 (95% CI 2.96–6.87), respectively. For other variables significantly associated with UL, the ORs were 0.9 (95% CI 0.82–0.99) for age at menarche, 1.70 (95% CI 1.22–2.38) for use of herbal medicine, 1.84 (95% CI 1.38–2.45) for family history of uterine fibroid, and 1.45 (95% CI 1.12–1.89) for miscarriage and/or abortion. Compared to participants with a dietary habit score of 0–35, those with scores 45–60 had OR for uterine fibroid of 0.66 (95% CI 0.46–0.95). The test for interaction was significant for the BET1L rs2280543 genetic variant and vegetarian diet (p for interaction = 0.046). After stratification (Table 3), the ORs among vegetarians and non-vegetarians with CT/TT genotype compared to the CC genotype were 0.77 (95% CI 0.57–1.06) and 0.15 (95% CI 0.05–0.47), respectively. Older age remained a significant risk factor for uterine fibroid among vegetarians and non-vegetarians whereas age at menarche, herbal medicine use, family history of uterine fibroid, and miscarriage/abortion were significant risk factors only among non-vegetarians. Using rs2280543 CC and non-vegetarian diet as the reference group (Table 4), the OR for uterine fibroid was 1.32 (95% CI 0.90–1.94) among vegetarians with the CC genotype, 0.78 (95% CI 0.57–1.07) among non-vegetarians with the CT/TT genotype, and 0.37 (95% CI 0.15–0.91) among vegetarians with the CT/TT genotype.

**Table 1:** Baseline characteristics of participants by vegetarian status

Variable	Non-vegetarian (n = 1734)	Vegetarian (n = 263)	*p* value
Uterine fibroid			0.472
No	1442 (83.16%)	214 (81.37%)	
Yes	292 (16.84%)	49 (18.63%)	
BET1L rs2280543 genotype			0.367
CC	1319 (76.0%)	202 (76.81%)	
CT	388 (22.38%)	54 (20.53%)	
TT	27 (1.56%)	7 (2.66%)	
Age (years)			0.020
30–39	660 (38.06%)	88 (33.46%)	
40–49	830 (47.87%)	149 (56.65%)	
50–59	244 (14.07%)	26 (9.89%)	
Alcohol intake			0.472
No	1688 (97.35%)	258 (98.10%)	
Yes	46 (2.65%)	5 (1.90%)	
Cigarette smoking			0.112
No	1633 (94.18%)	254 (96.58%)	
Yes	101 (5.82%)	9 (3.42%)	
Exercise			0.676
No	1231 (70.99%)	190 (72.24%)	
Yes	503 (29.01%)	73 (27.76%)	
Coffee intake			0.031
No	1053 (60.73%)	178 (67.68%)	
Yes	681 (39.27%)	85 (32.32%)	
Tea intake			0.636
No	1108 (63.90%)	172 (65.40%)	
Yes	626 (36.10%)	91 (34.60%)	
Hypertension			0.775
No	1585 (91.41%)	239 (90.87%)	
Yes	149 (8.59%)	24 (9.13%)	
Diabetes			0.956
No	1627 (93.83%)	247 (93.92%)	
Yes	107 (6.17%)	16 (6.08%)	
Hormone use			0.412
No	1595 (91.98%)	238 (90.49%)	
Yes	139 (8.02%)	25 (9.51%)	
Herbal medicine use			0.581
No	1505 (86.79%)	225 (85.55%)	
Yes	229 (13.21%)	38 (14.45%)	
Family history of uterine fibroid			0.271
No	1439 (82.99%)	211 (80.23%)	
Yes	295 (17.01%)	52 (19.77%)	
Miscarriage/abortion			0.302
No	687 (39.62%)	113 (42.97%)	
Yes	1047 (60.38%)	150 (57.03%)	
Fat intake score			0.004
0–35 (< Q1)	408 (23.53%)	53 (20.15%)	
36–40 (Q1–Q2)	443 (25.55%)	93 (35.36%)	
41–44 (Q2–Q3)	419 (24.16%)	64 (24.33%)	
45–60 (> Q3)	464 (26.76%)	53 (20.15%)	
Body mass index (kg/m^2^)	23.18 ± 3.50	23.61 ± 3.48	0.062
Menarcheal age (years)	13.35 ± 1.40	13.37 ± 1.35	0.806
Parity	2.09 ± 0.87	2.10 ± 0.92	0.898

Categorical data are presented as n (%) while continuous data are presented as mean ± SD

n, sample size; SD, standard deviation

BET1L rs2280543 CC genotype = wildtype, C = major allele, T = minor allele, minor allele frequency (MAF) = 0.128935

**Table 2:** Odds of uterine fibroids among participants

Variable	OR (95% CI)	*p* value
BET1L rs2280543 (ref: CC)		
CT/TT	0.69 (0.51–0.93)	0.014
Vegetarian (ref: No)		
Yes	1.09 (0.77–1.55)	0.617
Age (ref: 30–39 years)		
40–49	3.05 (2.20–4.21)	< 0.001
50–59	4.51 (2.96–6.87)	< 0.001
Alcohol intake (ref: No)		
Yes	1.34 (0.65–2.74)	0.425
Cigarette smoking (ref: No)		
Yes	1.03 (0.59–1.78)	0.924
Exercise (ref: No)		
Yes	1.08 (0.83–1.41)	0.586
Coffee intake (ref: No)		
Yes	1.15 (0.89–1.48)	0.294
Tea intake (ref: No)		
Yes	0.92 (0.71–1.20)	0.543
Hypertension (ref: No)		
Yes	1.27 (0.86–1.89)	0.236
Diabetes (ref: No)		
Yes	1.23 (0.78–1.94)	0.369
Hormone use (ref: No)		
Yes	1.23 (0.81–1.85)	0.328
Herbal medicine use (ref: No)		
Yes	1.70 (1.22–2.38)	0.002
Family history of uterine fibroid (ref: No)		
Yes	1.84 (1.38–2.45)	< 0.001
Miscarriage/abortion (ref: No)		
Yes	1.45 (1.12–1.89)	0.006
Fat intake score (ref:0–35)		
36–40	0.89 (0.64–1.26)	0.518
41–44	0.84 (0.59–1.19)	0.327
45–60	0.66 (0.46–0.95)	0.024
BMI	1.00 (0.97–1.04)	0.947
Menarcheal age	0.90 (0.82–0.99)	0.031
Parity	0.97 (0.84–1.12)	0.693

OR, odds ratio; ref, reference; BMI, body mass index; CI, confidence interval

**Table 3:** Risk of uterine fibroids stratified by vegetarian status

Variable	Non-vegetarian		Vegetarian	
	OR (95% CI)	*p* value	OR (95% CI)	*p* value
BET1L rs2280543 (ref: CC)				
CT/TT	0.77 (0.57–1.06)	0.112	0.15 (0.05–0.47)	0.001
Age (ref: 30–39 years)				
40–49	2.88 (2.03–4.07)	< 0.001	8.05 (2.78–23.27)	< 0.001
50–59	4.31 (2.76–6.74)	< 0.001	15.19 (3.34–69.09)	< 0.001
Alcohol intake (ref: No)				
Yes	1.42 (0.67–3.03)	0.360	1.57 (0.15–16.73)	0.707
Cigarette smoking (ref: No)				
Yes	0.99 (0.55–1.78)	0.977	1.35 (0.21–8.89)	0.755
Exercise (ref: No)				
Yes	1.15 (0.86–1.54)	0.338	0.64 (0.29–1.44)	0.279
Coffee intake (ref: No)				
Yes	1.14 (0.87–1.50)	0.346	1.18 (0.54–2.57)	0.674
Tea intake (ref: No)				
Yes	0.91 (0.69–1.21)	0.507	0.97 (0.44–2.12)	0.936
Hypertension (ref: No)				
Yes	1.25 (0.81–1.92)	0.313	1.58 (0.51–4.85)	0.427
Diabetes (ref: No)				
Yes	1.2 (0.74–1.96)	0.461	1.49 (0.4–5.64)	0.555
Hormone use (ref: No)				
Yes	1.37 (0.88–2.13)	0.160	0.5 (0.14–1.76)	0.283
Herbal medicine use (ref: No)				
Yes	1.64 (1.14–2.36)	0.007	2.42 (0.9–6.52)	0.080
Family history of uterine fibroid (ref: No)				
Yes	1.87 (1.37–2.55)	< 0.001	2.01 (0.86–4.70)	0.107
Miscarriage/abortion (ref: No)				
Yes	1.49 (1.12–1.98)	0.007	0.92 (0.43–1.96)	0.834
Fat intake score (ref:0–35)				
36–40	0.98 (0.68–1.41)	0.897	0.45 (0.17–1.20)	0.113
41–44	0.86 (0.59–1.26)	0.446	0.48 (0.16–1.39)	0.174
45–60	0.66 (0.45–0.97)	0.036	0.54 (0.18–1.59)	0.261
BMI	0.99 (0.95–1.03)	0.551	1.13 (1.01–1.27)	0.028
Menarcheal age	0.90 (0.82–0.99)	0.045	0.83 (0.62–1.10)	0.192
Parity	0.98 (0.84–1.15)	0.826	0.78 (0.51–1.19)	0.249

BET1L rs2280543*vegetarian diet, *p* value = 0.046

OR, odds ratio; ref, reference; BMI, body mass index; CI, confidence interval

**Table 4:** Risk of uterine fibroids based on BET1L rs2280543 genotypes and vegetarian status

Variable	OR (95% CI)	*p* value
BET1L rs2280543 genotype and vegetarian diet (ref: CC and non-vegetarian)		
CC and vegetarian	1.32 (0.90–1.94)	0.157
CT/TT and non-vegetarian	0.78 (0.57–1.07)	0.122
CT/TT and vegetarian	0.37 (0.15–0.91)	0.030
Age (ref: 30–39 years)		
40–49	3.08 (2.22–4.26)	< 0.001
50–59	4.58 (3.00–6.99)	< 0.001
Alcohol intake (ref: No)		
Yes	1.31 (0.64–2.69)	0.456
Cigarette smoking (ref: No)		
Yes	1.03 (0.59–1.79)	0.919
Exercise (ref: No)		
Yes	1.08 (0.83–1.41)	0.577
Coffee intake (ref: No)		
Yes	1.16 (0.90–1.50)	0.254
Tea intake (ref: No)		
Yes	0.92 (0.71–1.20)	0.550
Hypertension (ref: No)		
Yes	1.29 (0.87–1.93)	0.203
Diabetes (ref: No)		
Yes	1.23 (0.78–1.95)	0.364
Hormone use (ref: No)		
Yes	1.23 (0.82–1.86)	0.320
Herbal medicine use (ref: No)		
Yes	1.72 (1.23–2.40)	0.002
Family history of uterine fibroid (ref: No)		
Yes	1.87 (1.40–2.49)	< 0.001
Miscarriage/abortion (ref: No)		
Yes	1.42 (1.09–1.85)	0.009
Fat intake score (ref:0–35)		
36–40	0.88 (0.62–1.23)	0.450
41–44	0.82 (0.57–1.17)	0.263
45–60	0.66 (0.46–0.94)	0.021
BMI	1.00 (0.97–1.04)	0.880
Menarcheal age	0.90 (0.82–0.99)	0.026
Parity	0.97 (0.83–1.12)	0.639

OR, odds ratio; ref, reference; BMI, body mass index; CI, confidence interval

## Discussion

BET1L rs2280543 CT + TT genotype was significantly associated with a lower risk of UL among premenopausal Taiwanese vegetarians. Our results support previous findings on the involvement of BET1L rs2280543 polymorphism in UF pathogenesis [21]. So far, this is the first study to replicate the association between BET1L rs2280543 and UL among Taiwanese women. It is also the first study to demonstrate a significant interaction between BET1L rs2280543 and vegetarian diet on the risk of UF.

In addition to the identification of susceptible individuals [7, 18, 20, 21], insights into the molecular aspects of UL could assist in the development of targeted therapies. Several clinical management options for ULs are available and some are under investigation [33, 34]. Current treatment options for UL could be improved by incorporating the molecular information on UL. This might optimize treatment outcomes.

Profound knowledge of the lifestyle factors associated with UL could uncover non-invasive preventive and management options that could reduce UL-related surgical procedures and costs [2]. There is controversy regarding the impact of diet on UL risk [2, 9]. Notwithstanding, several studies have reported significant inverse associations between vegetarian diet and UL risk [8, 10–14]. The protective effect of vegetarian diet against UL is, in part accounted for by high fiber contents [10]. A low-fat and high-fiber diet (such as a vegetarian diet) affects the metabolism and activities of sex hormones and is believed to enhance fecal excretion of estrogen [35, 36]. Additionally, phytoestrogens (plant-derived estrogens) could reduce the risk of UL because they are believed to compete for estrogen receptors with estradiol [12]. Moreover, phytochemicals and antioxidants in vegetarian diet could enhance apoptosis [26, 37, 38] and inhibit estrogen metabolism [12, 37, 38]. It should be noted that estrogen levels are directly associated with the risk of UL [28, 39–42].

The biological role of BET1L rs2280543 predisposition to UL has been proven by its significant link with BET1L transcription and expression [21, 24]. Similar to our findings, BET1L rs2280543 was significantly associated with a lower risk of UL among Han Chinese [24] and European Americans [18, 23]. Although this SNP was first reported [21] and later replicated among Japanese [25], their findings differed from ours and other findings [18, 23, 24]. This could be due to discrepancies in the reference allele/genotype in the various studies. For instance, Sakai and colleagues used T as their reference allele while we and other authors used CC as the reference genotype. The association of BET1L rs2280543 and UL risk was not significant among black Americans [27] and Saudi women [26].

Delayed menarche was associated with a lower risk, while increasing age was associated with a higher risk of UL in our study, confirming previously published findings [5, 6, 27, 28, 42–45]. Other significant risk factors that we observed include, BMI, use of herbal medicine, family history, miscarriage, and abortion. These factors have also been previously reported [2, 45–53].

Consumption of vegetarian food rich in fats, such as oil-cooked vegetables could negate the health benefits of a vegetarian diet [54]. As such, we adjusted for this using the fat intake score and found that those who consumed foods relatively low in fat had a lower risk of UL. Therefore, in studies focusing on vegetarian diet, consideration of cooking methods and fat intake habits could help minimize errors in estimating the health benefits of vegetarian diet.

### Strengths and limitations

The large sample size is the strength of our study. However, since participation in the Taiwan Biobank project is restricted to individuals aged 30–70 years, we could not determine the association in premenopausal women aged below 30 years. As such, our results may not be generalized to all Taiwanese premenopausal women.

## Conclusions

In this study, BET1L rs2280543 CT + TT was associated with a lower risk of uterine fibroids, especially among vegetarians. These findings add to existing knowledge on the interplay between genetic and lifestyle factors in the development of UL, shedding more light on the epidemiology of the disease. From our findings, a vegetarian diet could prevent or reduce the risk of UL and, therefore, should be recommended.

## Supplementary Information

**Additional file 1: Table S1.** Questions pertaining to the frequency of eating fat-containing foods over the previous month.

## Data Availability

The data that support the findings of this study are available from Taiwan Biobank but restrictions apply to the availability of these data, which were used under license for the current study, and so are not publicly available. Data are however available from the authors upon reasonable request and with permission of Taiwan Biobank.
