# Supplementary material for: Risk of uterine leiomyoma based on BET1L rs2280543 single nucleotide polymorphism and vegetarian diet
Source: BMC Womens Health. 2022 Apr 27;22:139. doi: 10.1186/s12905-022-01721-1 (PMC9044639; doi:10.1186/s12905-022-01721-1)
Supplement: Supplementary file 1 — Additional file 1: Table S1. Questions pertaining to the frequency of eating fat-containing foods over the previous month. [file 12905_2022_1721_MOESM1_ESM.docx]

**Questions pertaining to the frequency of eating fat-containing foods over the previous month.**

| 1) Do you eat meat together with the skin? |
| --- |
| 2) Do you cook meat/fish with oil? |
| 3) Do you fry vegetables before eating? |
| 4) Do you eat noodles/rice with lard or fried vegetables? |
| 5) Do you fry bean products (tofu, bean curd) before eating? |
| 6) Do you spread cream, butter, or mayonnaise on bread before eating? |
| 7) When you eat snacks, do you choose to replace high-fat snacks (such as potato chips, pastries, doughnuts) with foods such as vegetables or fruits? |
| 8) Do you prefer braised or roasted meat? |
| 9) If a portion of food is a low-fat diet, do you choose to replace it? |
| 10) Do you try to replace high-fat meat (such as pork or beef) with low-fat meat (such as fish or chicken)? |
| 11) Do you choose to eat lean meat instead of pork belly or hooves? |
| 12) Do you eat light vegetarian food at certain meals to reduce the consumption of high-fat food such as meat? |
